# The Rationale for Consuming Cognitive Enhancement Drugs in University Students and Teachers

**DOI:** 10.1371/journal.pone.0068821

**Published:** 2013-07-17

**Authors:** Sebastian Sattler, Carsten Sauer, Guido Mehlkop, Peter Graeff

**Affiliations:** 1 Faculty of Sociology, Bielefeld University, Bielefeld, Germany; 2 Institute of Psychology, University of Freiburg, Freiburg, Germany; 3 Collaborative Research Center 882, Bielefeld University, Bielefeld, Germany; 4 Faculty of Economics, Law and Social Sciences, University of Erfurt, Erfurt, Germany; 5 Faculty of Humanities, Bundeswehr University Munich, Munich, Germany; The Scripps Research Institute, United States of America

## Abstract

Cognitive enhancement (CE) is the pharmaceutical augmentation of mental abilities (e.g., learning or memory) without medical necessity. This topic has recently attracted widespread attention in scientific and social circles. However, knowledge regarding the mechanisms that underlie the decision to use CE medication is limited. To analyze these decisions, we used data from two online surveys of randomly sampled university teachers (N = 1,406) and students (N = 3,486). Each respondent evaluated one randomly selected vignette with regard to a hypothetical CE drug. We experimentally varied the characteristics of the drugs among vignettes and distributed them among respondents. In addition, the respondent’s internalization of social norms with respect to CE drug use was measured. Our results revealed that students were more willing to enhance cognitive performance via drugs than university teachers, although the overall willingness was low. The probability of side effects and their strength reduced the willingness to use CE drugs among students and university teachers, whereas higher likelihoods and magnitudes of CE increased this propensity. In addition, the internalized norm against CE drug use influenced decision making: Higher internalization decreased the willingness to use such medications. Students’ internalized norms more strongly affected CE abstinence compared with those of university teachers. Furthermore, internalized norms negatively interacted with the instrumental incentives for taking CE medication. This internalization limited the influence of and deliberation on instrumental incentives. This study is the first to provide empirical evidence regarding the importance of social norms and their influence on rational decision making with regard to CE. We identified previously undiscovered decision-making patterns concerning CE. Thus, this study provides insight into the motivators and inhibitors of CE drug use. These findings have implications for contending with CE behavior by highlighting the magnitude of potential side effects and by informing the debate regarding the ethics of CE use.

## Introduction

### Cognitive Enhancement (CE) in Academia

CE can be defined as “the amplification or extension of [the] core capacities of the mind through improvement or [the] augmentation of internal […] information processing systems” ([1], p. 311). Healthy individuals may perceive CE drug use as a benefit-seeking strategy to enhance their cognitive abilities [2]–[4]. Potential cognitive enhancing medications include methylphenidate, (dextro-) amphetamine, donepezil, and modafinil [5], [6]. These drugs are prescribed as treatment for a variety of disorders, including attention deficit hyperactivity disorder (ADHD), postural orthostatic tachycardia syndrome, Alzheimer’s disease or dementia, shift work sleep disorder, and narcolepsy (e.g., [4], [7]).

Some authors predict that CE will be a forthcoming trend that will shape history and herald a 21st century of neuroscience [8]. One reason for this trend might be increasing pressure at work due to increased competition and workload [9]. Therefore, CE can be understood as an instrumental adaption to cope with these demands [4].

University students and teachers can be seen as populations at risk for CE drugs use [10], as success in academia depends on “brainpower”, and a need to perform at high levels may have increased due to competition over the last few decades (e.g., [11]).

The current prevalence rate of CE drug use is unknown. However, one poll found that 20% of the surveyed readers of Nature magazine [10] used drugs during their lifetime to augment performance. In another survey, 23% of physicians in North America were willing to use cognitive enhancers of proven efficacy if they were approved for use and had no severe associated risks [12]. For students, surveys suggest a lifetime prevalence of CE drug use ranging from 3% to 11% in the U.S. [13] and 0.7% to 4.5% in Germany [14], [15].

In addition, most studies only provide data concerning the prevalence and specific interactions with sociodemographic variables without referring to a theoretical model. Therefore, little is known regarding the decision-making process among CE users, and a theoretical framework guiding empirical investigations is needed [7], [16]–[18].

### The Mechanisms of Using Performance Enhancing Drugs

We refer to the self-medication hypothesis [19], [20], which proposes that individuals apply strategies to reduce their cognitive interference or compensate for certain deficits despite the potentially negative aspects of medication [21]. This non-formalized idea approximates the general approach of the classical Rational Choice Theory (RCT), which assumes that actors are utility maximizers who make decisions by weighing the pros and cons of possible action alternatives [22]–[24]. We adopt this idea to explain CE drug use (cf. [4]), as consuming performance-enhancing drugs is based on a decision-making process, which includes the following instrumental incentives: 1) the benefit of using CE medication to increase mental performance; 2) the probability of achieving this benefit; 3) the costs associated with CE drug consumption (i.e., the potential side effects); and 4) the probability of these costs.

The desired goal of CE drug use is to increase cognitive performance relative to an actual baseline state [1] by enhancing concentration, allowing students to study for more hours [25] or increasing working memory performance [26]. Boosting one’s self-esteem or the desire to improve one’s position relative to others in competition for prospective jobs and other assets [1], [27] might be additional reasons. Studies have shown that a low student grade point average and highly competitive admission criteria at colleges are associated with higher rates of the non-medical use of prescription stimulants [17]. This finding might indicatethat CE drug use is a strategy to attain success [15]. In addition, CE drugs might be a means to cope with stressors and increase personal performance [16].In general, the benefits of CE drug use are small-to-moderate for healthy individuals [1], [28], and a diminishing return can be expected [26], [29], [30].

However, the effects vary widely across individuals (e.g., [26], [29]–[33]), and high-performing individuals benefit the least from CE drugs [29], [30], [34]. Consequently, the desired benefits occur with a certain likelihood.

For healthy individuals, concerns exist regarding the potential side effects and long-term health consequences of CE drug use (e.g., fatal arrhythmias, excitotoxicity, emesis, sexual dysfunction, addiction, depression, sleep difficulties, reduced appetite and weight loss as well as weight gain, hypertension, headaches, high blood pressure, and even changes in personality; [21], [27], [30], [34]–[37]). These costs may outweigh the benefits [5]. Counterfeit medication purchased on the Internet is particularly associated with risks as well as unintended overdosing by self-medication [26], [34] and risks emerging from the interactions of these drugs with other medications [37]. These negative consequences of off-label use are largely unknown for healthy individuals [5], [10]. Following the self-medication hypothesis, actors might be willing to accept the negative effects of a substance in exchange for the chance to achieve a desirable state [10], [19]. Some evidence shows that expected side effects of CE drugs reduce the frequency of their use [15]. However, additional research is needed concerning the extent to which side effects influence the decision-making process, as little is known about how respondents react to hints or information regarding drug characteristics [28].

These side effects do not occur with certainty, but they do occur with a specific probability. For example, Wezenberg et al. [38] found that 6 out of 10 healthy users of ampakine farampator (a treatment of Alzheimer’s disease and schizophrenia that can be used as a CE drug) suffered from headaches. This type of information is outlined in instructional leaflets for medically prescribed drugs; however the validity of such information for healthy users is unclear. Therefore, individuals must evaluate the likelihood of side effects before engaging in self-medication [27]. Rejecting or verifying the assumption of “naive users” will improve our understanding of the mechanisms that underlie CE drug use [39].

In addition to the variables considered by classical RCT, the normative dimensions of CE drug use (cf. [8], [28], [40]) must be explored for the following reasons: CE drugs might be perceived as an unfair means to gain advantages relative to others, which may place pressure on non-users to also use drugs [1], [21] and may infringe on others’ freedom of choice [8]. Furthermore, unequal access (e.g., because of financial restrictions) can violate the norms of fairness [3]. In addition, the violation of authenticity norms has been discussed via the implication that “native or achieved excellence has a higher worth than talent that is bought” ([1], p. 326). Using medication for another purpose might be socially prohibited or taboo and can be regarded as a socially undesirable abuse of drugs. Actually, little is known about how potential users make decisions with regard to this normative dimension [28].

According to Hechter and Opp [41], social norms can be defined as moral imperatives (i.e., social actions that should (or should not) be processed without taking in account consequences for the actor) or more generally as guidelines for individual actions in the absence of moral imperatives. We propose that the social norms that prohibit CE drug use have not been internalized to the same degree across all actors.

Some scholars argue that following or violating a norm can be regarded as rational behavior [42], [43]. Although breaking a norm can result in internal penalties such as psychological costs, following an internalized norm can result in intrinsic rewards [43], [44]. Several studies have provided evidence that norms are crucial determinants of behavior (e.g., [45], [46]). Considering internalized norms as psychological incentives breaks with the traditional assumption in RCT that actors hold preferences only for tangible or “hard” incentives [47]. Extending RCT models using the concepts of social psychology is helpful in predicting behavior [48]. A person’s willingness to take CE drugs is expected to decrease as internalized norms against CE use become stronger.

Other scholars posit that social norms should not be regarded as a part of a rational decision-making process [49], [50], i.e., by prescribing behavior in unconditional and non-outcome-oriented ways [51]–[53]. If a potential action is classified as “wrong” due to a particular norm, then this action is more likely to be removed from the agenda. Therefore, norms function as filters for non-appropriate alternatives (also see [54], p. 75). Thus, deliberation no longer becomes necessary, and benefits are weighted down, completely ignored, or the deterring effect of the costs increases [52], [55]. Actors often rely on proven and well-known strategies without much consideration of alternate strategies (e.g., a rule of thumb or social norm) to reduce the costs of in-depth deliberation [52], [55]. The stronger the internalized norm, the less individuals deliberate on the costs and benefits of CE drug use, and the more likely individuals are to refrain from their use.

The single elements of the decision-making process according to classical RCT can be summarized in an expected utility term (U = q * B – p * C) where *q* is the probability that the drug works, *B* is the increase in performance, *p* is the probability that side effects will occur, and *C* is the severity of side effects (cf. [53], [56], [57]). Our first hypothesis states that an increase in expected utility from CE drug use increases the probability of CE drug use (*H1*). Second, when individuals internalize social norms that are contrary to CE medication use, the probability of using CE drugs decreases (*H2*). Third, the effects of internalized norms and expected utilities will interact in the following manner: When the norms are strongly internalized, the effect of utility on the probability of CE drug use will decrease (*H3*).

### The Present Study

To understand the decision-making process regarding the use of CE drugs, and to test the above hypotheses, we conducted two surveys. Although pioneering studies often used small, non-randomized and selective samples [58], [59], our large-scale surveys use randomized samples of students and an exhaustive survey of university teachers. Within the context of a vignette study, participants rated their willingness to take hypothetical CE drugs, which were experimentally varied with regard to 1) increases in mental performance and 2) the likelihood of experiencing increases in mental performance, 3) potential side effects, and 4) the probability of experiencing side effects. Furthermore, participants assessed their internalized norms to abstain from CE drug use. By analyzing the role of instrumental incentives, internalized norms, and the interaction between the two, we gained knowledge concerning the mechanisms that underlie the decision-making process with respect to CE drugs.

## Methods

### Participants

The empirical tests of the hypotheses were based on two large random samples of university students (N = 5,048) and university teachers (N = 3,980). We provide the information suggested by the Checklist for Reporting Results of Internet E-Surveys (CHERRIES). A three-stage random design was applied using self-administered online surveys for students conducted in February 2011: First, four German universities were selected; second, academic disciplines were chosen; and third, students were randomly sampled. The same universities were used for the sample of university teachers, and all university teachers of the randomly chosen disciplines were contacted. A total of 3,486 students and 1,402 university teachers responded to the survey. The student response rate was 69.1%, and the teacher response rate was 35.3%. The completion rates of these groups were 93.1% and 77.2% for students and teachers, respectively; these rates are similar to those of previous studies [17], [18], [60]–[62]. The analyses were conducted on 3,209 students and 1,064 university teachers due to dropouts and incomplete responses. Females comprised 60.9% of the student sample and 31.1% of the teacher sample. The median ages of students and teachers ranged between 22–23 years and 36–40 years, respectively.

### Data Quality Assurance

The survey procedure provided full anonymity to ensure privacy and obtain unbiased results (see ethics statement below). Participation was voluntary. Participants received a pre-notification letter in the mail that contained information concerning the purpose of the study, data security, and the conditions of participation. The letter was followed by an e-mail invitation and up to two reminders. E-mail invitations contained a personal password-protected link that prevented multiple participations. We incentivized our participants to obtain a higher participation rate and better data quality (e.g., [63]). Every student could choose a reward (EUR 5 via the mail or a PayPal account, a voucher for an online store, or one out of two receipts of charitable donations). Academic faculty could choose from EUR 5 on a PayPal account, a voucher for an online store or two receipts of charitable donations. All instruments passed an extensive expert pretest procedure. Furthermore, we conducted think-aloud pretests to ensure the validity of our measures. The usability and technical functionality of the survey tool (EFS Survey 8.0) were also tested. Our instruments were presented on two pages, with the vignette on one page and the questionnaire on internalized social norms on another page.

### Experimental Setting and Vignette Construction

We used a factorial survey approach [64], [65] to test the theoretical assumptions (studies that use vignettes, see [66], [67]; for general hypothetical CE use, see [12], [68]). The factorial survey described decisional situations (so-called vignettes) across several attributes (dimensions) that varied experimentally in their levels. The vignettes described a fictitious university student (or teacher) with the opportunity to use a drug to enhance his cognitive abilities for studies (or work) without medical necessity. We employed a factorial survey approach because it allowed us to vary all level combinations experimentally, and this design is more immune to socially desired answering than direct questioning due to its hypothetical character [69]–[71]. The vignettes provided information on cognitive benefits and the costs in terms of side effects (i.e., headache) as well as the probabilities that these benefits and costs would occur (see Table 1). Costs and benefits varied systematically by size and the probability of occurrence. To illustrate the dimension “magnitude of headache” as a side effect, we used the Wong-Baker FACES Pain Rating Scale (Copyright Wong-Baker FACES Foundation, www.WongBakerFACES.org; used with permission), which is a commonly used, highly valid and reliable method to measure pain severity [72]–[74]. The scale appeared under the vignette text but was omitted in vignettes that did not include side effects. We used all possible vignette combinations (N_vignettes_ = 1,080, full factorial design) and added the 36 combinations of q and B without side effects, thereby yielding 1,116 vignettes in total. Each respondent was randomly assigned to one vignette; each vignette was rated approximately three times on average (min = 1; max = 5). The wording of the vignettes was as follows (examples in brackets):

**Table 1:** Vignette Dimensions, Levels, and Coding in Both Studies.

Dimension	Levels	Coding
Probability ofperformance increase (q)	1/20/40/60/80/100 percent	0.01/0.2/0.4/0.6/0.8/1
Increase of mentalperformance (B)	1/50/100/150/200/250 percent	0.01/0.5/1/1.5/2/2.5
Probability ofheadache (p)	No side effects/1 out of 100,000 users/1 out of 10.000 users/1 out of 1,000users/1 out of 100 users/1 out of 10 users/every user	0/0.00001/0.0001/0.001/0.01/0.1/1
Magnitude ofheadache (C)	[No side effects]/slight headache (value 2 on the scale)/moderate headache(value 4 on the scale)/strong headache (value 6 on the scale)/very strongheadache (value 8 on the scale)/extreme headache (value 10 on the scale)	0/0.2/0.4/0.6/0.8/1
	We displayed the Wong-Baker FACES™ 10-point pain scale that uses visual(faces with various degrees of pain), verbal (no hurt, hurts little bit, hurts littlemore, hurts even more, hurts whole lot, hurts worst), and numerical cues.

*“The next questions focus on a very recent topic. Please read the next paragraph carefully and imagine the following situation: A university teacher* {version for students: “a student”} *considers enhancing his cognitive performance for his work* {version for students: “studies”} *by using a prescription drug without any medical necessity. Someone can provide him with the pills for free. A study that found that the drug increases cognitive performance by* [250] *percent with a likelihood of* [60] *percent caught his attention. In addition, the side effects were investigated: Using this medication causes* [slight] *headaches (value* [2] *on the scale) for one out of* [100,000] *users. Additional side effects are unknown.”*

Using the question, “Would you consume the drug if you were in his position?”, respondents applied a 10-point Likert-scale to rate the likelihood of use from “strongly against use” (0) to “strongly in favor of use” (9). The number of missing responses was low: 13 (0.4%) students and 39 (2.7%) university teachers did not respond.

### Norm Measure

The process of norm internalization involves social learning and conditioning (i.e., via sanctions) [75] such that after successful internalization, actors follow norms without monitoring or sanctioning. To measure the degree to which social norms are internalized, we referred to the Theory of Normative Internalization [76], which explains how an internalized social norm becomes a moral commitment. Our operationalization of morals is similar to measures used in prior studies [45], [77], [78]. University students and teachers specified their respective moral commitment with regard to the use of prescription drugs without medical necessity using the following question: “How do you personally evaluate using prescription drugs to enhance studying [for university teachers, working] performance without any medical necessity? I think this use is …”. Evaluations were recorded using a 7-point rating scale that ranged from “absolutely moral” (1) to “absolutely not moral” (7). Items for university teachers were “…when one must work intensively on something important”; “…during an appointment that is decisive for ones work”; and “…generally for work”. Items for students were “before an examination”; “during an examination”; and “in general for university studies”. In each study, a weighted sum score based on the three items above was calculated. Scale reliability coefficients were satisfactory (alpha_students_ = 0.95; alpha_university teachers_ = 0.96).

### Ethics Statement

The ethical principles as formulated in the WMA Declaration of Helsinki guided our research project. If research objectives do not refer to issues regulated by law (e.g., the German Medicine Act [Arzneimittelgesetz, AMG], the Medical Devices Act [Medizinproduktegesetz, MGP], the Stem Cell Research Act [Stammzellenforschungsgesetz, StFG] or the Medical Association’s Professional Code of Conduct [Berufsordnung der Ärzte]), then no ethics approval is required for social science research in Germany. Our study has no such objectives; therefore, no approval was required. Paragraph 28 of the Data Protection Act of North Rhine Westphalia (Datenschutzgesetz Nordrhein-Westfalen, DSG NRW) states that personal data should be used anonymously; furthermore, it states that participant consent is only required when the data are not anonymous. All data were anonymous. Participation was voluntary, and people who opted not to participate or withdrew were not penalized. Anonymity and informed consent to use the data were communicated and obtained through a declaration of data security and included in all communications. Anonymity was ensured given that a) the research team did not have access to names or addresses (e-mail or postal); b) the cooperating universities had no data access; and c) we used secure sockets layer (SSL) protocols. The legal services of Bielefeld University approved all procedures. After informing respondents of these mechanisms, participation was understood as being conclusive action. An official data protection officer supervised our project and data collection.

## Results

**Figure 1:**
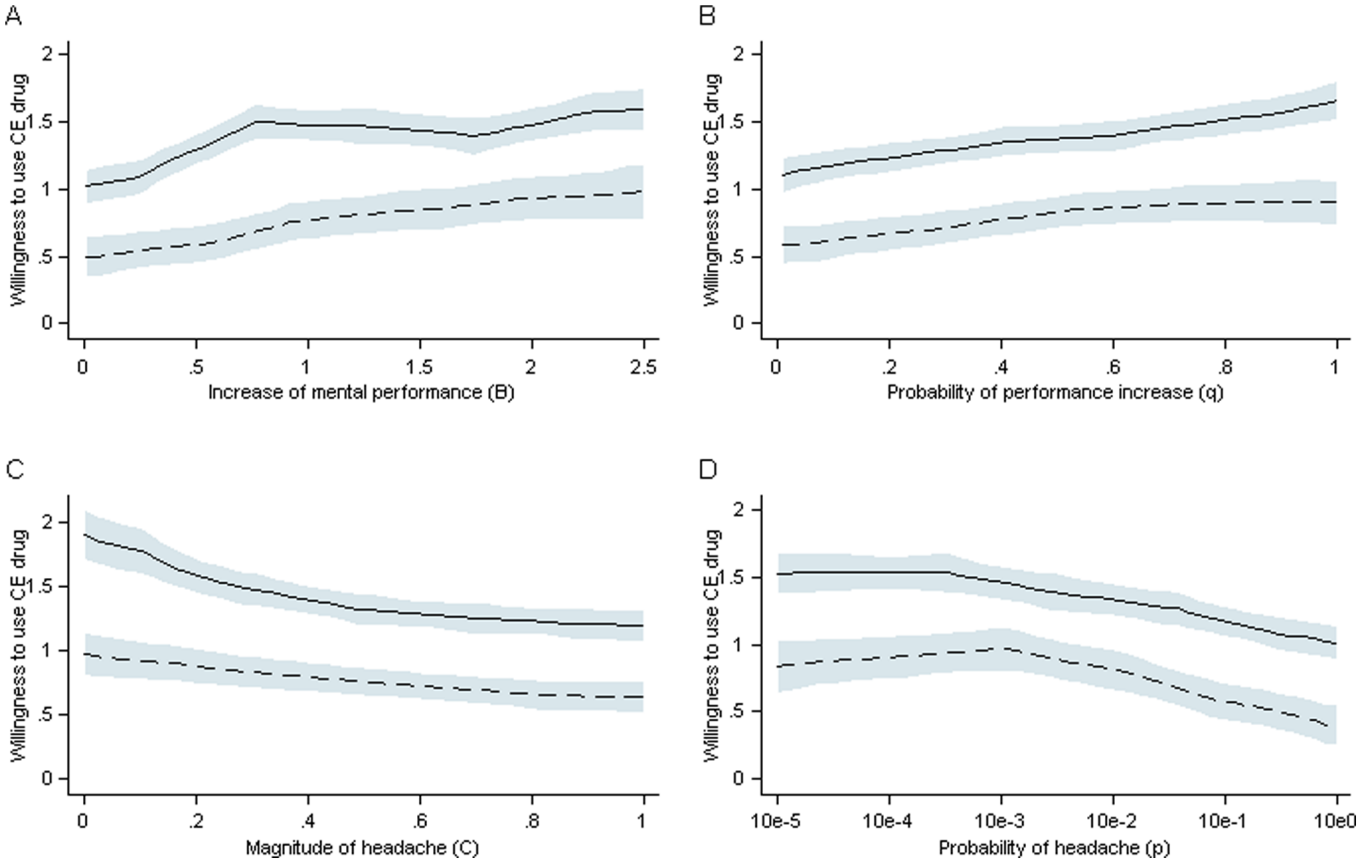
Willingness of Students and Teachers to Use Cognitive Enhancers with Four Varying Characteristics. Each panel shows the mean willingness to use a CE pill on a 10-point Likert scale with the following anchors: 0 = “strongly against use” and 9 = “strongly in favor of use”. Corresponding confidence intervals with local polynomial smoothing are also shown. The lines in each panel indicate a generally lower willingness to use CE drugs for university teachers (dashed lines) compared with students (drawn lines). Mean willingness increases with increases in mental performance (A). Mean willingness increases as the probability of performance increases (B). Mean willingness decreases as the magnitude of headache increases (C). Mean willingness decreases as the (logarithmized) probability of headache increases (D).

First, students (mean = 1.38; sd = 0.04) showed a greater willingness to take the cognitive enhancers described in the vignettes than university teachers (mean = 0.77; sd = 0.05; t = −9.05; p<0.001). This difference is shown in Figure 1: All of the lines that represent students are located above those for university teachers.

Second, Figure 1 shows the association between the vignette dimensions. As the performance-enhancing effect of the CE drug (i.e., the RCT benefits [*B*]) increased, respondents were more willing to take the drug. The ascending lines indicate this effect in Panel A. In addition, increases in the probability of a performance increase (*q*, Panel B) increased this willingness. Conversely, as the magnitude of a headache (i.e., RCT costs [*C*, Panel C]) and the probability of its occurrence (*p*, Panel D; we transformed *p* using its base-10 logarithm to place it within the range of the other variables) increased, the willingness to take CE pills decreased.

Third, these effects were tested in multivariate models (see Models 1 and 2 in Table S1) in which we simultaneously tested for the effect of internalized social norms [the norm internalization of students (mean = 5.58; sd = 1.73) was lower than that of university teachers (mean = 5.23; sd = 1.82). The latter variable showed the expected negative effect on the willingness to take CE drugs in both populations (p_students_<0.001 and p_university teachers_<0.001, see Models 1 and 2). University students and teachers with a stronger internalized norm against CE drug use were less willing to use these drugs.

**Figure 2:**
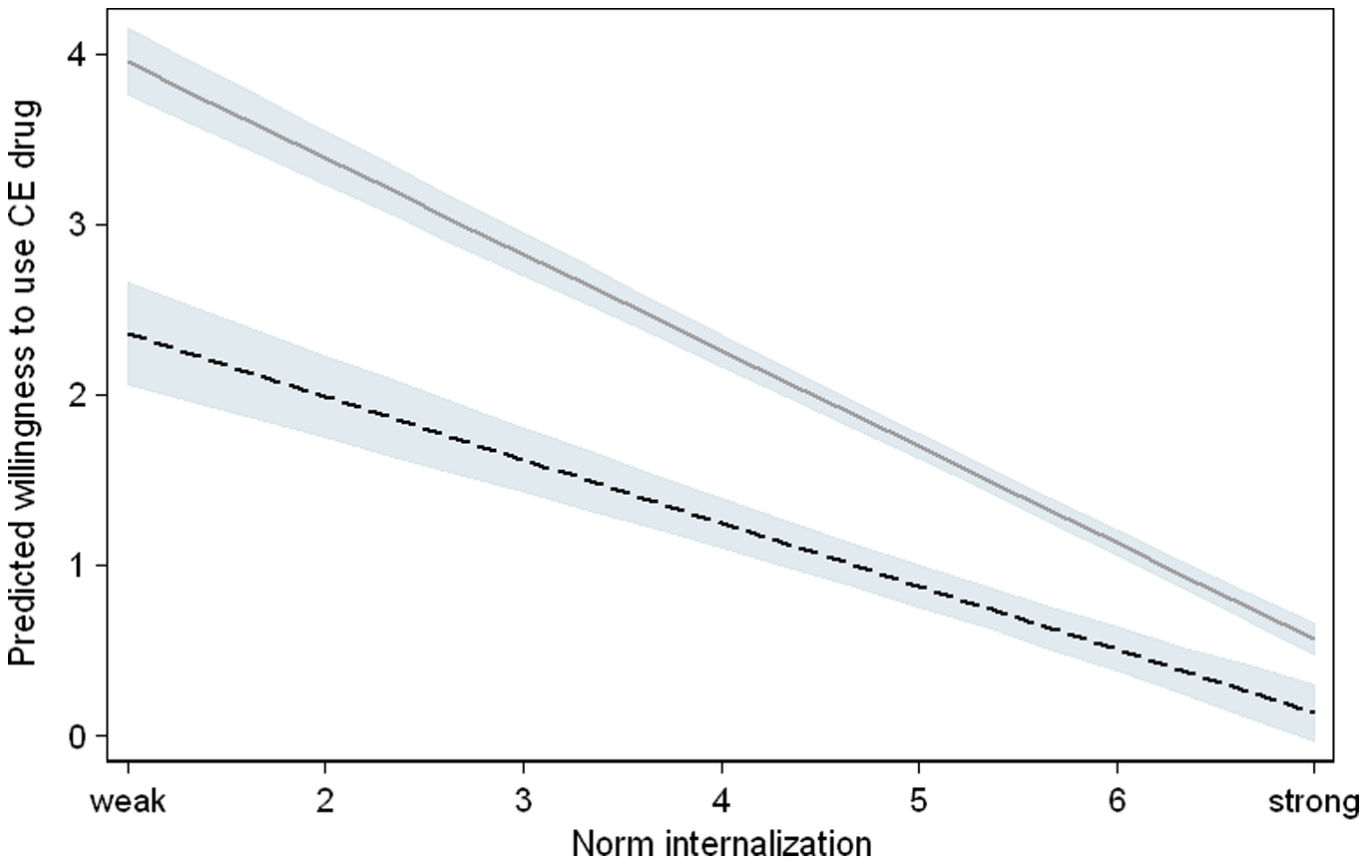
Willingness of University Students and Teachers to Use Cognitive Enhancers Depending on Their Norm Internalizations. The estimated mean willingness to use a CE pill was measured on a 10-point Likert scale with the following anchors: 0 = “strongly against use” and 9 = “strongly in favor of use”. Corresponding confidence intervals based on Model 4 (all effects with the exception of norm internalization and the group variable were set to their mean values) are also shown. Mean willingness decreased as a function of norm internalization. This decrease was stronger in university students (light grey line) than teachers (black line).

To validate the previously described differences in the willingness to use CE drugs, we used a pooled dataset of university students and teachers and included a dummy variable to indicating the population (see Model 3). Again, the positive coefficient (p<0.001) revealed that students were more willing to use these drugs than university teachers. Group comparison tests were employed to test whether university students and teachers reacted in the same way to the drug characteristics described by the vignette dimensions and whether their norm internalization worked equally (Model 4). These goals were accomplished by computing the interaction effects between each vignette dimension and the population type. The treatments influenced both types of participants similarly because significant interactions did not exist for the vignette dimensions. However, we found an interaction effect for the norm variable (p<0.001): The same level of norm internalization had a stronger negative effect on students’ willingness to use CE drugs compared with university teachers (see Figure 2).

Fourth, classical RCT assumes that actors consider all four instrumental determinants because individuals do not respond to benefits and costs independent from their respective probabilities of occurrence (i.e., even a great benefit will not motivate people to take a drug when the probability of the effects is approximately zero (see [79], p.39). Therefore, a composite utility measure (*U*) was constructed by subtracting the weighted costs (p*C) from the weighted benefits (q*B). In both studies, the effect of *U* (see Models 1 and 2 in Table S2) revealed a significant (p_students_<0.001 and p_university teachers_<0.001) positive effect with regard to the willingness to use the CE drug. Thus, a pill that produced a higher performance boost while having lower side effects led to a greater willingness to use a CE drug. This finding is in line with the classical RCT. Nevertheless, the norm effect remained stable. Models 3 and 4 replicated the findings from Table S1: We found a) a greater willingness among students to use CE drugs; b) no differences with regard to the different effects of the vignette dimensions on utility across populations (the insignificant interaction between *U* and the grouping variable in Table S2 indicates this result); and c) a stronger effect of internalized norms in students.

**Figure 3:**
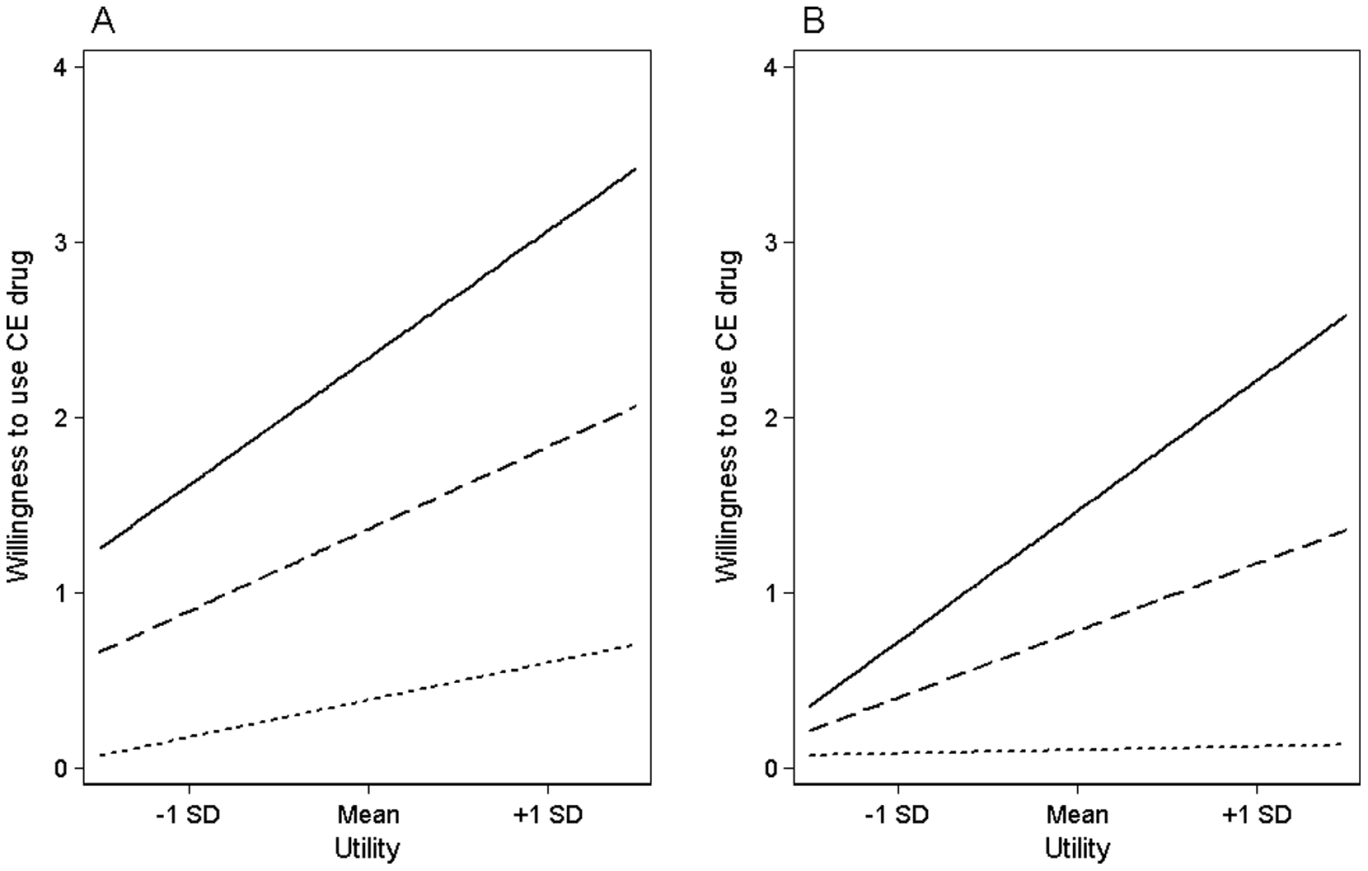
Willingness of Students and Teachers to Use Cognitive Enhancers Depending on Utility, Internalized Norms, and Interactions. Each panel shows the mean willingness to use a CE pill on a 10-point Likert scale with the following anchors: 0 = “strongly against use” and 9 = “strongly in favor of use”. A strong norm internalization (dotted lines), leads to a flat slope, which indicates that utility does not increase the willingness of university students (Panel A) or teachers (B). A weak internalization (drawn lines) leads to steep slopes, which indicates that utility strongly increases this willingness. A medium internalization (dashed lines) leads to a moderate ascent of the slopes.

Fifth, our extended RCT postulated that stronger norm internalization would decrease the effect of utility. We found this negative interaction effect in both populations (p_students_<0.001; p_university teachers_<0.001, see Models 1 and 2 in Table S3). This effect did not differ between students and teachers (see the interaction of *U*, *N*, and the grouping variable in Model 4 in Table S3). As the internalization of the social norm against CE use strengthens, the effect of the utility decreases the willingness to use the drug. This effect can be seen in Figure 3, which is based on Table S3. An increasing slope indicates the increasing influence of utility. The strength of norm internalizations modulated this influence: For university students and teachers with strong internalizations (see the dotted lines in Panels A and B), the slope of the lines was approximately parallel with the x-axis; thus, utility plays virtually no role. Conversely, the slopes were steep in case of a weak internalization (see the drawn lines); thus, utility shows a strong positive effect. A medium internalization (see the dashed lines) moderately reduced the ascent of the slopes.

## Discussion

We aimed to understand and explain the mechanisms underlying prescription drug use to enhance cognitive performance among university students and teachers. We assumed that the use of CE drugs results from a decision-making process driven by instrumental incentives and internalized social norms. In the following section, we summarize and interpret our findings, discuss limitations, and postulate the implications of the study.

### Summary and Interpretation of Results

Our study is among the first to use large random samples of students and teachers to assess their willingness to use CE drugs. While the overall willingness to use CE drugs was low, we found that students were more willing than teachers to engage in CE drug use.

The findings in both populations support the theoretical assumptions of our modified rational-choice model: An increase in utility boosts the probability of using CE medication. During decision making, actors consider benefits and risks and are therefore instrumentally oriented (cf. [4], [52]). This process indicates that users are neither naive nor exclusively benefit oriented. Consequently, *H1* was supported.

To the best of our knowledge, this is the first study using an extended RCT model which shows that the decision to take CE drugs is not only based on rational deliberation but also influenced by internalized social norms. For some people, drug use without any medical indication (i.e., an identified illness) might be seen as normatively dubious. Furthermore, some people might perceive conflicts with social norms such as rules of fairness or authenticity [1], [40]. The results endorse the hypothesis (*H2*) that deeply internalized norms against CE drug use significantly reduce the willingness to use them, even if their utility is controlled. This finding can be explained by the fact that a violation of internalized norms can result in internal penalties such as psychological costs (e.g., [43], [44], [46]). We found that students are more sensitive to internalized norms than university teachers, suggesting that stronger internalization led students to more strongly refrain from using CE drugs.

Our findings also support the assumption of an interaction between utility and internalized social norms (*H3*) [52], [53], [80]. As internalized norms against CE drug use increased, the effect of utility on the decision to take CE medication was reduced. Actors deliberated less on the potential utility of CE drugs. Instead, the strong internalized norms overruled the instrumental incentives of using CE medication as an alternative. Strongly internalized norms work as a filter to refrain from using drugs without deliberation [54], [81], [82].

### Study Limitations

One limitation of this study is the relatively low response rate among university teachers. However, with regard to other studies in this population [61], [62] and the high opportunity costs for participation among university teachers, the achieved rate was satisfactory.

Furthermore, this study was conducted only in Germany. Several factors that could result in different consumption patterns across countries (e.g., drug availability, acceptability of CE drug use, legal status of CE substances, prices, and culturally accepted strategies to achieve success) should be investigated in further multi-country studies.

Because university students and teachers have a special demand for cognitive abilities, they might have a greater need for CE medication than other people. In addition, further studies should be conducted to determine whether our results are generalizable to other settings in which alertness, vigilance, or high cognitive performance matter, especially in fields that involve continuous productivity or repetitive work. Therefore, future studies may apply our model to the general population with the goal of replicating our results. However, Iit is intriguing that two groups in different life stages showed similar decision-making patterns even though they differed in their opportunity structure and costs. On the one hand, university teachers might be more motivated to use CE drugs, as competition among university teachers might be stronger than the competition among students. On the other hand, university teachers might have already invested a great deal into their career. Therefore, they might have more to lose from the social disapproval that results from (detected) CE misuse [83].

Future research could also complement the findings of our study using an extended RCT model by embedding additional factors in the theoretical framework (e.g., by considering variables such as peer prevalence, personal experience, the price of CE drugs, personal characteristics, etc.).

In this study, we investigated only the use of pharmaceutical CE drugs. The use of other CE methods (see [1], [84]–[86]), such as transcranial direct current stimulation, education, mental training, genetic modifications, prenatal enhancement, non-prescription drugs or illegal drugs such as cocaine, requires further research. In addition, we did not investigate other reasons for which people choose to use CE pharmaceuticals, such as mood lifting, weight loss, getting high, etc. [7], [18], nor did we explore the type of the participants’ pharmaceutically enhanced cognitive function that participants might experience. It must also be noted that different substances (e.g., methylphenidate, atomoxetine, modafinil, or mixed amphetamine salts) vary in their desired and adverse effects. These differences might influence the decision-making process. However, one strength of our experimental design is that multiple combinations of wanted and adverse effects, which might be representative of certain classes of medication or specific substances, were investigated. By varying the drug class and the affected type of cognitive functioning, further studies could offer additional insights.

### Implications

Our extended RCT model enhance understanding of the decision-making process of individuals with respect to CE drug use. Healthcare professionals, physicians, universities, politicians, and others can use this knowledge and our findings to regulate CE drug use and create recommendations for policy and practice from empirical research. These findings highlight the fact that people consider the normative dimension of CE drug use in addition to its side effects and benefits.

In general, society, politics, and legislation must determine whether CE drug use should be legal and under what conditions its use should be considered appropriate; this also applies for universities [28]. These decisions are striking because CE drug use implies potential health risks but can be used to attain better personal outcomes that can also result in positive societal effects such as research innovations. This benefit is especially true in academia but is also true for other fields in which “brainpower” is an important determinant of success. We found that the internalized norms concerning CE drug use differed among respondents and that these norms were a crucial element in choosing to consume CE drugs. Regulations, such as laws or recommendations, that influence the emergence or alteration of internalized norms concerning CE drug use might be a way for policymakers to influence drug consumption. One way to regulating CE drug use among students could be mentioning CE drug use in university honor codes. These codes define what is right and wrong, explain sanctions, raise the awareness of students and university teachers concerning cheating, etc. (e.g., [87]). Research shows that a) faculty at institution with honor codes are more likely to use judicial procedures and established procedures for handling cheating incidents [87]; and b) students expect stronger sanctions from faculty at institutions with such codes [87], show less dishonesty, and rate their acceptance and understanding of the academic integrity policy higher [87]–[89]. Research is needed to investigate whether CE drug use is considered to be cheating, as our study only demonstrates that a certain number of students have internalized social norms against CE drug use as well as how students would react to the regulation of CE drugs in their universities. This topic should be addressed in future research. However, norm internalization not only exerts negative main effects on drug use but also decreases the tempting effect of higher utility when deciding whether to take CE drugs. This mechanism strengthens the power of the drug regulations that are intended to shape internalized norms.

Drug regulations are even more important as long as the health-related consequences are unclear. Some scholars have raised strong concerns because they fear serious physical or psychiatric problems, especially when CE medication is not under medical supervision [35]. Side effects may cause an increased demand on the public healthcare system, which is ultimately a political issue. A more liberal position states that the decision to use medication should be up to competent individuals “as an expression of autonomy” ([3], p. 53); however, they should also be responsible for any negative effects [40]. The deterrence effect of side effects found in our study can be translated into policy (potential) users with more reliable information concerning these adverse effects and by providing alternative strategies to help individuals reach their desired goals [1], [4]. However, potential prevention and intervention strategies must take the varying severities and types of side effects of different drugs into account (e.g., psychological side effects such as addiction or physiological side effects such as fatal arrhythmias). In addition, to avoid exaggerated expectations among some individuals with respect to the benefits of CE drugs, it should be communicated that, based on current knowledge of the available drugs, the effects of such drugs are small-to-moderate for healthy individuals [28], [33].

## Supporting Information

Table S1 * p<0.05, ** p<0.01, *** p<0.001. Table S1 shows the OLS coefficients (and robust standard errors) of the willingness to use CE drugs on the four vignette dimensions, internalized norms, and population type. Model 1 contains the vignette dimensions and the norm measure for the student sample. Increasing the probability and strength of cognitive performance increased the willingness to use CE drugs, whereas the greater probability and strength of headaches and stronger norm internalization decrease such likelihood. Model 2 shows similar effects for university teachers. Model 3 shows that students are more likely to use CE. Model 4 shows that the negative effect of norm internalization is stronger for students, but the vignette dimensions have an equal effect on the evaluation in both populations.
(DOCX)

Table S2 * p<0.05, ** p<0.01, *** p<0.001. Table S2 shows the OLS coefficients (and robust standard errors) of the willingness to use CE drugs on the utility of drug use and the norms across university students and teachers study. Model 1 shows the positive effect of utility and the negative effect of internalized norms on students. Model 2 shows similar effects for university teachers. Model 3 shows that the willingness to use CE drugs is greater for students. Model 4 shows that the utility effect is similar in both studies, but the negative impact of internalized norms is greater for students.
(DOCX)

Table S3 * p<0.05, ** p<0.01, *** p<0.001. Table S3 shows the OLS coefficients (and robust standard errors) of the willingness to use CE drugs on utility of drug use, internalized norms, and their interaction across university students and teachers. Model 1 shows the positive effect of utility and the negative effect of internalized norms on students. It also shows that the effect of utility decreases when norm internalization increase and a negative interaction effect. Model 2 shows similar effects for university teachers. Model 3 shows that the willingness to use CE is greater for students. Model 4 shows that all effects are similar across population groups (with the exception of the negative effect of internalized norms, which is stronger for students).
(DOCX)
