# Supplementary material for: The Rationale for Consuming Cognitive Enhancement Drugs in University Students and Teachers
Source: PLoS One. 2013 Jul 17;8(7):e68821. doi: 10.1371/journal.pone.0068821 (PMC3714277; doi:10.1371/journal.pone.0068821)
Supplement: Table S1 — * p<0.05, ** p<0.01, *** p<0.001. Table S1 shows the OLS coefficients (and robust standard errors) of the willingness to use CE drugs on the four vignette dimensions, internalized norms, and population type. Model 1 contains the vignette dimensions and the norm measure for the student sample. Increasing the probability and strength of cognitive performance increased the willingness to use CE drugs, whereas the greater probability and strength of headaches and stronger norm internalization decrease such likelihood. Model 2 shows similar effects for university teachers. Model 3 shows that students are more likely to use CE. Model 4 shows that the negative effect of norm internalization is stronger for students, but the vignette dimensions have an equal effect on the evaluation in both populations. (DOCX) [file pone.0068821.s001.docx]

|  |  |  |  |  |
| --- | --- | --- | --- | --- |
|  | ***(1)***  ***Students***  b/se | ***(2)***  ***University teachers***  b/se | ***(3)***  ***Total***  b/se | ***(4)***  ***Total***  b/se |
| Probability of Performance Increase (q) | .552*** | .494*** | .549*** | .494*** |
|  | (.108) | (.141) | (.089) | (.141) |
| Increase of Mental Performance (B) | .246*** | .216*** | .235*** | .216*** |
|  | (.042) | (.057) | (.035) | (.057) |
| Probability of Headache (p) | -.607*** | -.530*** | -.587*** | -.530*** |
|  | (.089) | (.106) | (.073) | (.105) |
| Magnitude of Headache (C) | -.684*** | -.513** | -.642*** | -.513** |
|  | (.130) | (.176) | (.107) | (.176) |
| Internalized Norm (N) | -.972*** | -.677*** | -.899*** | -.677*** |
|  | (.050) | (.069) | (.041) | (.068) |
| Students (=1; Teachers=0) |  |  | .580*** | .626*** |
|  |  |  | (.062) | (.190) |
| Students*q |  |  |  | .058 |
|  |  |  |  | (.178) |
| Students*B |  |  |  | .031 |
|  |  |  |  | (.071) |
| Students*p |  |  |  | -.078 |
|  |  |  |  | (.138) |
| Students*C |  |  |  | -.171 |
|  |  |  |  | (.219) |
| Students*N |  |  |  | -.296*** |
|  |  |  |  | (.085) |
| Constant | 1.291*** | .665*** | .698*** | .665*** |
|  | (.116) | (.151) | (.102) | (.150) |
| R-squared | .209 | .183 | .211 | .215 |
| Respondents | 3209 | 1064 | 4273 | 4273 |
