# Supplementary material for: The Rationale for Consuming Cognitive Enhancement Drugs in University Students and Teachers
Source: PLoS One. 2013 Jul 17;8(7):e68821. doi: 10.1371/journal.pone.0068821 (PMC3714277; doi:10.1371/journal.pone.0068821)
Supplement: Table S2 — * p<0.05, ** p<0.01, *** p<0.001. Table S2 shows the OLS coefficients (and robust standard errors) of the willingness to use CE drugs on the utility of drug use and the norms across university students and teachers study. Model 1 shows the positive effect of utility and the negative effect of internalized norms on students. Model 2 shows similar effects for university teachers. Model 3 shows that the willingness to use CE drugs is greater for students. Model 4 shows that the utility effect is similar in both studies, but the negative impact of internalized norms is greater for students. (DOCX) [file pone.0068821.s002.docx]

|  |  |  |  |  |
| --- | --- | --- | --- | --- |
|  | ***(1)***  ***Students***  b/se | ***(2)***  ***University teachers***  b/se | ***(3)***  ***Total***  b/se | ***(4)***  ***Total***  b/se |
| Utility (U) | .477^***^ | .387^***^ | .456^***^ | .387^***^ |
|  | (.052) | (.072) | (.043) | (.087) |
| Internalized Norm (N) | -.974^***^ | -.680^***^ | -.901^***^ | -.680^***^ |
|  | (.037) | (.050) | (.030) | (.061) |
| Students (=1; Teachers=0) |  |  | .578^***^ | .532^***^ |
|  |  |  | (.070) | (.087) |
| Students*U |  |  |  | .090 |
|  |  |  |  | (.100) |
| Students*N |  |  |  | -.294^***^ |
|  |  |  |  | (.070) |
| Constant | 1.123^***^ | .591^***^ | .556^***^ | .591^***^ |
|  |  |  | (.062) | (.190) |
| R-squared | .199 | .167 | .200 | .204 |
| Respondents | 3209 | 1064 | 4273 | 4273 |
