# Supplementary material for: The Rationale for Consuming Cognitive Enhancement Drugs in University Students and Teachers
Source: PLoS One. 2013 Jul 17;8(7):e68821. doi: 10.1371/journal.pone.0068821 (PMC3714277; doi:10.1371/journal.pone.0068821)
Supplement: Table S3 — * p<0.05, ** p<0.01, *** p<0.001. Table S3 shows the OLS coefficients (and robust standard errors) of the willingness to use CE drugs on utility of drug use, internalized norms, and their interaction across university students and teachers. Model 1 shows the positive effect of utility and the negative effect of internalized norms on students. It also shows that the effect of utility decreases when norm internalization increase and a negative interaction effect. Model 2 shows similar effects for university teachers. Model 3 shows that the willingness to use CE is greater for students. Model 4 shows that all effects are similar across population groups (with the exception of the negative effect of internalized norms, which is stronger for students). (DOCX) [file pone.0068821.s003.docx]

|  |  |  |  |  |
| --- | --- | --- | --- | --- |
|  | ***(1)***  ***Students***  b/se | ***(2)***  ***University teachers***  b/se | ***(3)***  ***Total***  b/se | ***(4)***  ***Total***  b/se |
| Utility (U) | .469^***^ | .383^***^ | .449^***^ | .383^***^ |
|  | (.052) | (.071) | (.043) | (.086) |
| Internalized Norm (N) | -.842^***^ | -.497^***^ | -.756^***^ | -.497^***^ |
|  | (.045) | (.061) | (.037) | (.075) |
| U*N | -.256^***^ | -.363^***^ | -.284^***^ | -.363^***^ |
|  | (.051) | (.070) | (.043) | (.086) |
| Students (=1; Teachers=0) |  |  | .575^***^ | .531^***^ |
|  |  |  | (.070) | (.087) |
| Students*U |  |  |  | .087 |
|  |  |  |  | (.100) |
| Students*N |  |  |  | -.345^***^ |
|  |  |  |  | (.086) |
| Students*U*N |  |  |  | .106 |
|  |  |  |  | (.099) |
| Constant | 1.122^***^ | .591^***^ | .558^***^ | .591^***^ |
|  | (.046) | (.062) | (.065) | (.075) |
| R-squared  Respondents | .205 | .187 | .209 | .212 |
|  | 3209 | 1064 | 4273 | 4273 |
